# Des Préparations D’argent Et De Leur Utilité Dans Le Traitement Des Maladies Vénériennes

**Published:** 1839-11-23

**Authors:** 


### BIBLIOGRAPHICAL NOTICES.

"*Des Préparations d'argent et de leur utilité dans le traitement des maladies Vénériennes.* Par Adrien Sicard, *Docteur en Médecine*, &c. &c. Montpellier: 1839. 8vo., pp. 84."

*On the Preparations of Silver, and their Efficacy in the Treatment of Venereal Diseases.* By Adrien Sicard, M. D., &c. &c. Montpelier: 1839. 8vo., pp. 84.

An impartial review of the Essay of Mr. Serre published on the subject under consideration, and an analysis of several cases treated by silver, which we collected in the wards of Mr. Biett, had led us to a conviction of the inefficacy of the salts of silver in the treatment of syphilis. We could not avoid the conclusion that it was in fact an inert substance. Beyond an irritation of the alimentary canal, no symptom appeared after the administration of this supposed remedy, either in the twenty-five cases of Mr. Serre, or in those published by Dr. Trudeau and myself, that could be attributed to the effects of silver on the animal economy. Notwithstanding these impressions, we commenced the perusal of the essay of Mr. Sicard with, we think, a perfect openness to conviction, and a determination to give due weight to the clinical facts presented, if in number and accuracy of statement they could claim superiority over those which we had published on the subject. A careful perusal of this essay, however, so far from inducing us to surrender our opinions, has confirmed and strengthened the conclusions which we had previously arrived at. To do full justice to the work of Mr. Sicard, so far as the practical portion of it is concerned, and as the topic is of considerable therapeutic interest, we propose to make a brief analysis of the essay, and put the reader in possession of the facts, for the exercise of his own judgment.

We pass over the first part, which is devoted to a review of the work of Mr. Serre, to commence at once with the analysis of the cases.

Obs. 1st. "*Chancre on the prepuce.*"—The patient, a man, entered the Hôtel Dieu St. Eloi, of Montpelier, sixteen days after the first appearance of the disease, having up to the time submitted to no treatment. He presented upon the prepuce "a chancre as large as a ten sous piece, the edges perpendicular, the surface of the sore very red, and but little painful;" (venesection, rest.) On the twenty-first day of the disease, Mr. Serre ordered the chloride of silver gr. 1-10th daily; no local application. A few days later, dried lint was placed between the prepuce and the glans penis. On the fifteenth day of the treatment, in the place of the chancre, there was a red and indurated spot, which soon became very considerable. The local application of the powder of Vienna was now added to the treatment. About three weeks later, the patient was ordered to take two glasses of sarsaparilla daily. In a few days the induration was much reduced, and sulphur ointment was then applied to the parts. The patient left, cured, fifty-seven days after his entrance, and seventy three days from the first appearance of the chancre.

Obs. 2d. "*Chancres at the base of the gland.*"—This patient, a soldier, of a lymphatic temperament, and of a feeble constitution, treated himself during one month by the sole application of simple cerate. At his entrance there existed, "at the base of the glans, a chancre, six lines long, and three wide; edges perpendicular; surface of the ulcer very red;" (pills of chloride of silver, dry charpie locally.) On the fortieth day of the treatment the chancre was cicatrized, but there remained an "indurated point," for which the powder of Vienna was ordered, and the bichloride of mercury gr. $\frac{1}{8}$, daily. The patient finally left, preserving still "a little induration." Duration of treatment sixty-one days.

Obs. 3d. "*Chancres encircling the base of the gland.*"—A soldier, twenty-three years of age, of of a lymphatic temperament. At his entrance, (twenty days after the commencement of the ulceration,) he presented, at the base of the glans penis, "a chancre which surrounded it in the form of a collar, six lines in breadth; edges perpendicular; surface of ulcer dark red, covered with a grayish matter." After a repose of ten days, Mr. Serre ordered the chloride of silver gr. 1-10th; no local application. In twenty-seven days the chancre cicatrized; there remained no induration.

Obs. 4th. "*Chancres at the base of the gland.*"—The patient, a soldier, twenty-five years of age, of a lymphatico-sanguine temperament, and of a feeble constitution. Date of the commencement of the disease not given. During the first sixteen days which followed the entrance of the patient, thirty pills of Sédillot (strong mercurial ointment,) were administered without producing any effect, except a slight diminution in the quantity of the suppuration. At this period there existed at the base of the glans, near the frenum, "a chancre of the size of a ten sous piece, very painful, and suppurating abundantly; pus grayish, and very fœtid." One pill of chloride of silver gr. 1-10th daily; dry lint to the ulcer. After four pills the pain had entirely ceased, and the suppuration had sensibly diminished. On the twenty-sixth day after the administration of the silver the chancre had cicatrized.

Obs. 5th. "*Bubo in the right groin—inoculation—chancre in the thigh.*"—The patient, a soldier, twenty-six years of age, of a feeble constitution, and of a lymphatic temperament, entered the ward six days after the appearance of a bubo in the right groin. The day after his entrance the bubo was opened. Inoculation on the thigh with the pus of the bubo, produced a chancre. Chloride of silver, in pill; emollient cataplasms to the ulcer. The chancre was cured in about thirty days.

Obs. 6th. "*Chancre and bubo.*"—Subject, a soldier, twenty-four years of age, of a lymphatic temperament, and of a feeble constitution. Ten days after the commencement of the disease, the patient presented a chancre upon the prepuce "as large as a piece of ten sous, and a bubo as large as a chicken's egg. Chloride of silver internally; application of the salt of ammoniac to the bubo; dry lint to the chancre. Three days later, fifteen leeches around the bubo, followed by emollient cataplasms; in a few days the bubo was opened. The chancre was cicatrized on the twenty-eighth day; but the bubo was not healed until the sixty-seventh day, "owing to the dressing being badly applied, and giving rise to fistulæ."

Obs. 7. "*Two vegetations in the form of a bridle at the base of the gland.*"—G., a soldier, twenty-two years of age, of a feeble constitution. Before his entrance the vegetations had been cut off and cauterized, but they returned, accompanied by an engorgement, which prevented the patient from uncovering the glans; his only internal treatment had been a tisan, with sarsaparilla. After a few baths of mallows, the glans could be uncovered; two vegetations were then seen—the largest, six lines long, and five lines at the base, was situated near the frenum; the second had the volume of a pea; they were both of a bright red colour. Chloride of silver, one pill daily. The vegetations became paler; two pills daily were then given; shortly after, the deuto-chloride of mercury gr. 1-10th, daily, was added. Under this treatment the vegetations became pale, and a "little diminished" in size. Local baths, with the liquor of Van Swieten, were next added. The baths, according to the author, produced a good effect. The patient left the wards entirely cured; duration of treatment seventy-six days.

Obs. 8th. "*Enormous cauliflower excrescence at the corona glandis.*"—This patient, a soldier, twenty-two years of age, of a bilious temperament, and of a strong constitution, was attacked with a discharge from the urethra, after which two chancres made their appearance, but soon healed; these were followed in their turn by a large cauliflower excrescence, which, placed at the base of the glans, partly covered this organ; it was composed of a large number of vegetations—was an inch in length; besides, there existed four warts upon the prepuce; the whole was of a scarlet colour. (Chloride of silver gr. 1-10th; general bath; local baths, with an infusion of mallows; no local application.) In forty-three days one of the warts fell off, and the vegetations were diminished in size. The vegetations had entirely fallen off on the fifty-sixth day. The dose of the silver was increased, as there remained some induration. The patient left the hospital, perfectly cured, on the ninety-second day.

Having thus given an analysis of all the cases, before proceeding to examine them individually, we wish to make a few preliminary remarks. We regret to say that these observations are so imperfect, that it is impossible, at times, to feel positive that the case under examination is actually a chancre. The diagnosis of this disease is a much more difficult point than the author of the essay seems to imagine, inasmuch as ulcerations on the genital organs, of a perfectly benign character, frequently occur from excoriations,—uncleanliness and acrid secretions causing the vesicles of a herpes preputialis, or an erythema of the genitals, (intertrigo,) to degenerate into an ulcer, &c. &c. Nor is it sufficient to have an accurate description of an ulcer itself: its size, form, depth, the state of the edges, surface, and particularly the existence or non-existence of induration at the base, are important. But, before one can judge of the severity of the disease, it is essential that the condition of the surrounding parts be stated, whether they are red, swollen, œdematous, &c., and this the author has neglected in every instance. A knowledge of the general state of the patient during the treatment, is equally indispensable; and it is not sufficient to confine ourselves to the statement that the patient is of a strong or a weak constitution, a lymphatic or a sanguine temperament. There are two points in the history of chancre which should constantly be borne in mind, but which Mr. S. has entirely neglected, in arriving at his conclusions. One of them is a truth beyond contradiction, to wit, that a chancre may cicatrize when left to itself. How often, in fact, in large venereal hospitals, do we see patients present themselves with a secondary eruption, and, on examining the genital organs, find an indurated spot, where a chancre has but recently cicatrized, often too in spite of the most absurd remedies which have been employed. This induration, then, which accompanies the true Hunterian chancre, we regard, in the second place, as the disease, or, at least, an important part of it, which it is indispensable to remove before the patient can be pronounced cured. Others, and perhaps with justice, go still farther, and regard this induration as an evidence that the constitution is affected. "It is," says Dr. Ricord, "the thermometer by which I judge of the existence and degree of the general infection: to remove this, then, by the knife, is of no avail, as the constitution would still remain tainted,—and, besides, such a proceeding would deprive us of its aid in making our prognosis, and in regulating our treatment." To prevent the induration, is, then, the first thing which should claim the attention of the physician. For this purpose, the usual and most rational treatment is to cauterize the chancre as early as possible with the nitrate of silver, (no existing circumstance contraindicating its employment,) in order to destroy the specific character of the disease, while the cauterization of the surrounding vessels diminishes the probability of absorption. The next object on the part of the physician is to promote the cicatrization by such local applications as the nature of the ulcer may require. At this period, as has been recommended by Wallace, mercury is often given to hasten a cure, and this indeed when fears of a general contamination are no longer entertained. But, let the induration once exist, and we then regard an internal treatment as necessary, in order to neutralize the properties of the virus, so to speak, and to promote the absorption of the indurated tissue: the mere cicatrization of an ulcer which may co-exist with the induration, we regard as of secondary importance.

After these general reflections, which we deem necessary for a proper appreciation of our remarks on Mr. Sicard's essay, we return to the examination of each of the above cases individually.

*Case 1st.*—The chancre was cicatrized in fifteen days after the commencement of the treatment with silver. This speedy cure will appear less remarkable, when we take into consideration the fact that the ulcer already dated thirty-six days back, when the remedy was first given; and that it is not impossible that the period of reparation had already commenced. The description of the chancre, we think, renders this the more probable, though its physical characters are not sufficiently given to enable us to pronounce positively. Supposing it to be otherwise, the cure was by no means completed, as there still existed a "considerable induration,"—to remove which it had been necessary to resort to the powder of Vienna, sarsaparilla, and sulphur ointment. The actual duration of the treatment becomes fifty-seven days, instead of fifteen. This, then, is far from being a case of speedy cure; and if it were, so complicated was the treatment, that with every disposition to do justice to the silver, we cannot appreciate its effects in distinction from those of the other remedies.

The same remarks may be applied to the second case. Here the chancre was cicatrized on the fortieth day,—but the induration remaining, the powder of Vienna, and the bichloride of mercury, were resorted to. The third case is less objectionable. It would have been important, however, to have known what transpired during the ten days which elapsed between the day on which the actual state of the patient was given, and that on which the treatment was commenced. Did repose produce no effect? Here the duration of the disease was only twenty-seven days; but the ulcer, it must be remembered, was unaccompanied by induration, and for such a case the cure is by no means remarkable. It must be recollected that when a new remedy is under notice which many facts seem to prove to be inert, an individual case must be very striking before we admit the relation between the supposed cause and the effect.

The fourth case requires a serious consideration. During sixteen days, thirty pills of Sédillot (pills of mercurial ointment) were given, without producing any other effect than a slight diminution in the quantity of pus secreted. The chloride of silver was then immediately given; the patient had no sooner taken 4-10ths of a grain than the pain, which had before been very acute, ceased entirely, and the suppuration became sensibly diminished. Can it be possible that so minute a dose could have produced so wonderful an effect, when others have given it in doses of several grains, without producing the slightest change either in the general or local condition of their patients? or was it rather the first remedy which had but just commenced to act upon the system, for not a day was permitted to elapse between the administration of the one and the other?

We do not think that either of the internal remedies produced this happy change; we attribute it solely to the application of the dry lint. During the administration of the pills there was no local application, consequently the pus was allowed to remain upon the surface which secreted it, which is at all times the worst practice; but no sooner was the chloride of silver ordered, than we remark the local application of the charpie. This we consider as amply sufficient to account for the diminution in the quantity of the suppuration, and the cessation of the pain. In fact, dry lint acts in these cases like a sponge, in absorbing the pus, which, suffered to remain, becomes acrid, decomposes, and invariably aggravates the local symptoms; and, besides, the gentle, stimulating effect of the charpie, tends to modify the character of the ulcer.

These few remarks, we think, are sufficient to show how short-sighted Mr. S. has been in neglecting to take into account the effect of so important an adjuvant, while he attributes the entire cessation of an acute pain, and the diminution of an abundant suppuration, to 4-10ths of a grain of chloride of silver. The duration of the disease from the change of treatment was twenty-six days. It may be well to remark here, that though Mr. Sicard repeats, in nearly every observation, "no local application," we find, nevertheless, that dry lint was applied to the majority of the chancres. This may be of trifling import in the eyes of Mr. S., but most physicians will consider it a very useful application.

In the fifth case, the chancre produced by inoculation from the bubo, required thirty days for its cure. In the sixth case, the duration of the chancre was about the same; but the bubo was not healed before the lapse of sixty-seven days. The seventh testifies neither pro nor con, as the treatment was too complicated, (chloride of silver, deuto-chloride of mercury, local baths, with the liquor of Van Swieten.)

In the eighth, the silver required ninety-two days to effect a complete cure,—fifty-six before the vegetation had fallen,—a period by no means remarkable for its short duration. Thus it will be seen that the average duration of the chancres in the above cases was twenty-eight days; and this, it must be remembered, only relates to their cicatrization. As regards the induration, the most important feature of the disease, the silver proved in every case perfectly useless,—the powder of Vienna, bichloride of mercury internally, and mercurial baths, having been resorted to by Mr. Serre himself, (in whose service these cases were taken,) before a cure could be effected. As for the effect of silver in the cases of buboes and vegetations, the duration of the treatment was longer than that which is generally required, and in one case failed entirely.

The perusal of this paper, together with what we have already published on the same subject, (see Examiner, vol. i. p. 348,) must, we think, bring conviction to the mind of every one that the numerous observations thus far brought forward prove the salts of silver to be substances, harmless when given in small quantities, irritating the alimentary canal when too largely given, and possessing no curative properties.

We have already so far extended our remarks, that we pass over that portion of the essay which relates to the examination of the cases of Mr. Ricord ineffectually treated by silver, leaving the reader to judge himself of their value. (See work of Mr. Ricord, p. 253.) We cannot but regret, however, that the trivial objections urged by Mr. S. against these cases, should have proceeded from one who professes to view this subject with an unprejudiced eye. It is inconsistent enough to say that Mr. R. did not cure his cases because he gave the silver in larger doses than Mr. Serre had recommended, as if the latter could regulate the dose of a remedy which gives no evidence of its action on the system appreciable to our senses. Still more absurd is it for him to declare that the action of the remedy was interfered with by his cauterizing some of the chancres, while elsewhere he blames Mr. Ricord for not repressing the granulations by the nitrate of silver. It is evident from Mr. Sicard's remarks on the cases of Mr. R., that as a pupil of Mr. Serre, he is but too eager to force upon the profession the assertions of the latter, contradicted as they are by the very observations which are brought to support them. W. P. J.

*Paris*, October 14th, 1839.

